# Molecular Surveillance, Prevalence, and Distribution of Cacao Infecting Badnavirus Species in Côte d’Ivoire and Ghana

**DOI:** 10.3390/v16050735

**Published:** 2024-05-06

**Authors:** George A. Ameyaw, Koffié Kouakou, Mohammed Javed Iqbal, Luc Belé, Valentin L. F. Wolf, Cory V. Keith, Bolou A. Bolou Bi, Christophe Kouamé, Donald Livingstone, Owusu Domfeh, Ebenezer A. Gyamera, Jean-Philippe Marelli, Judith K. Brown

**Affiliations:** 1 Cocoa Research Institute of Ghana, P.O. Box 8, New Akim-Tafo, E/R, Ghana; gaakumfi@yahoo.co.uk (G.A.A.);; 2 The Centre for International Forestry Research and World Agroforestry (CIFOR-ICRAF), Côte d’Ivoire Country Program, Cocody, Abidjan 08 BP 2823, Côte d’Ivoire; 3 School of Plant Sciences, 1140 E. South Campus Dr., The University of Arizona, Tucson, AZ 85721, USA; 4 Mars Wrigley Plant Science Laboratory, 434 G Street, Suite 200, Davis, CA 95616, USA

**Keywords:** *Badnavirus*, *Caulimoviridae*, cacao swollen shoot disease, epidemiology, mealybug vector, *Theobroma cacao*

## Abstract

The cacao swollen shoot disease (CSSD) caused by a complex of badnavirus species presents a major challenge for cacao production in West Africa, especially Ghana and Côte d’Ivoire. In this study, CSSD species detection efficiency, diversity, and geographic distribution patterns in cacao plantations in Ghana and Côte d’Ivoire were investigated through field surveillance, PCR detection assays, sequencing of positive amplicons, and phylogeographic clustering. Cumulatively, the detection efficiency of the tested CSSD primer sets that were targeting the movement protein domain of the virus ranged from 0.15% (CSSD-3 primer) to 66.91% (CSSD-1 primer) on all the symptomatic cacao leaf samples assessed. The identified CSSD species differed phylogenetically and overlapped in distribution, with the cacao swollen shoot Togo B virus (CSSTBV) (*n* = 588 sequences) being the most prevalent and widely distributed compared to the other CSSD species that were encountered in both countries. Geographically, the cacao swollen shoot CE virus (CSSCEV) species (*n* = 124 sequences) that was identified was largely restricted to the bordering regions of Ghana and Côte d’Ivoire. These results provide updated knowledge of the geographic distribution of the key CSSD species and their diagnostic efficiency and, thus, provide guidance in identifying locations for structured testing of cacao germplasm and optimal diagnostics for the predominant CSSD species in Ghana and Côte d’Ivoire.

## 1. Introduction

Cacao swollen shoot disease (CSSD) persists as a major cocoa production constraint throughout West Africa, where much of the world’s bulk cocoa is produced. The disease occurs widely in Côte d’Ivoire and Ghana, whose economies depend heavily on cultivation of the tree *Theobroma cacao* (L.), the source of beans used to manufacture chocolate and other confectionaries [1,2]. Since the initial outbreak in Ghana during the mid-1930s, the disease has spread to all West African cacao-growing regions [3,4] and led to the loss of several million cacao trees either due to tree death or by tree removal programs designed to minimize virus spread [3]. Swollen shoot disease affects cacao plants of all growth stages and symptom phenotype and severity can vary depending on the virus species involved, soil nutrition, and availability of shade or not at time of infection [4,5,6,7,8]. At least 14 mealybug species are capable of transmitting CSSD-associated badnaviruses. Transmission occurs in a semi-persistent manner [9,10]. The most economically important mealybug species are *Formicococcus njalensis* (Laing) and *Planococcus citri*, primarily because of high transmission efficiency and their widespread distribution in the cacao landscape [9,10,11]. The disease is known to be caused by a complex of badnaviruses belonging to the genus *Badnavirus* and family *Caulimoviridae* [8,12,13,14].

The CSSD badnavirus species endemic to Africa have a circular, double-stranded DNA (dsDNA) genome ranging in size from 7.0 to 9.2 kilobase pairs (kbp) and are encapsidated in a nonenveloped bacilliform particle that is 120–150 in length × 28–30 nm in diameter [4,5,15]. The complete genome sequence encodes five predicted open reading frames, ORFs 1, 2, 3 X and Y, on the plus strand [5]. Para-retroviruses, including the CSSD badnaviruses, replicate through a dsRNA molecule that is reverse transcribed from the viral genome. The badnavirus genome is transcribed to produce a greater-than-genome-length terminally redundant pre-genomic RNA, from which viral proteins are translated, or serves as the template for replication of the viral genome through reverse transcription [16]. Badnaviruses have been shown to evade DNA methylation and gene silencing to counter the plant host defenses [16]. 

Further characterization of the CSSD badnavirus species with modern molecular tools has resulted in the identification and documentation of several badnavirus species as the causal pathogen(s) of the cacao swollen shoot disease throughout West Africa [5,8,17,18]. Those associated with field infections of cacao in West Africa include cacao swollen shoot Côte d’Ivoire D virus (CSSCDV), cacao swollen shoot Côte d’Ivoire E virus (CSSCEV), cacao swollen shoot Ghana M virus (CSSGMV), cacao swollen shoot Ghana N virus (CSSGNV), and cacao swollen shoot Ghana Q virus (CSSGQV), cacao swollen shoot Togo A virus (CSSTAV), cacao swollen shoot Togo B virus (CSSTBV), and cacao swollen shoot Togo C virus (CSSTCV) [5,6,8,18,19,20]. The relationship between the geographic distribution of CSSD badnaviral species diversity is, however, not well understood. Despite advances made in the development of PCR amplification diagnostic tests using specific and/or degenerate primer pairs for CSSD detection [7,8], there exist gaps in knowledge about the spatial distribution and phylodynamics of CSSD badnaviruses. Also, PCR detection in symptomatic trees can be unreliable and virus may be undetectable despite the CSSD-like foliar symptoms [8,21,22]. These challenges underscored the need for an improved understanding of the prevalence and distribution of the recognized CSSD badnavirus species and for improved molecular tools to facilitate early disease detection. 

The objective of this study was to estimate disease prevalence and determine the geographical and spatial distribution of CSSD badnavirus species on cacao farms in the major production areas of Côte d’Ivoire and Ghana. To achieve this objective, CSSD badnavirus detection was carried out by polymerase chain reaction (PCR) amplification using four previously validated primer pairs, CSSD 1–4. The primers were designed to amplify fragments of different sizes of the movement protein (*mp*) encoding region in the open reading frame 3 (ORF3) of each respective CSSD species [8,22]. To establish provisional species identification, the amplicons were cloned, sequenced, and subjected to phylogenetic analysis with CSSD badnavirus reference *mp* sequences extracted from complete genome sequences available in GenBank. Provisional identification was based on clustering of isolates on the phylogenetic tree with their closest isolate at the species level. Reference species were demarcated according to the criteria by the International Committee on Taxonomy of Viruses (ICTV), which recognizes the cut-off of >80% pairwise nucleotide identity in reverse-transcriptase RNAse H genomic region for badnavirus as species demarcation criteria. The highly divergent *mp* genomic region has proven useful for establishing provisional species identification, based on sequence comparisons with the analogous *mp* fragment of well-characterized CSSD species for which a complete genome sequence is available [22]. The geographical and spatial distribution of CSSD badnavirus species infecting symptomatic cacao trees in the main cacao production areas of Côte d’Ivoire and Ghana were documented to gain a better understanding of the intra-species phylodynamics and lend insight into the potential for spread of each species to apparently virus-free cacao-growing areas throughout the region and to map the potential origin of diversification of five predominant CSSD species. The results of this study, which provides extensive mapping of CSSD species in both countries, will enhance effective disease management based on early disease detection. That, in turn, will be used to make informed decisions on removal and replacement of infected trees with virus-free and CSSD-tolerant cacao genotypes for improved productivity and sustainability of commercial cocoa production in West Africa.

## 2. Materials and Methods

### 2.1. Collection Sites and Cacao Plant Samples 

**Figure 1:**
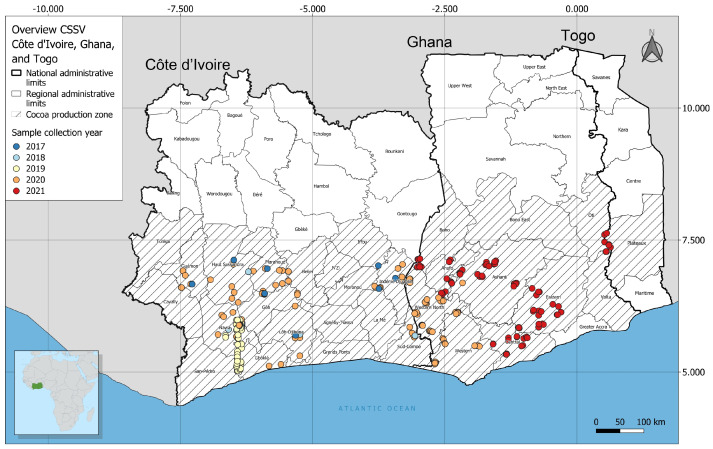
Map of the representative sample collection sites in Côte d’Ivoire and Ghana where symptomatic leaf samples from infected cacao trees were progressively collected across the selected cacao plantations for the study.

Symptomatic leaf samples were purposively and continuously collected from representative CSSD-infected cacao plantations in Côte d’Ivoire and Ghana and progressively updated between 2017 and 2023 for molecular diagnostics, field mapping, data analysis, and phylogenetic clustering (Figure 1). In Côte d’Ivoire, the collection sites were selected based on the locations in the main cacao-producing areas where widespread CSSD outbreaks had been previously reported (Figure 1) [19]. In Ghana, leaf samples were collected from farms where previous outbreaks had been documented that spanned the Ahafo, Ashanti, Bono, Central, Eastern, Oti, Volta, Western–north and Western–south cocoa regions. Global positioning system (GPS) co-ordinates were recorded at each collection site (Figure 1). A total of 674 symptomatic cacao trees were sampled. Several flush leaves were collected from each tree and placed into a 50 mL screwcap tube containing 100% glycerol. The tubes were placed on ice in a Styrofoam cooler and transported to the laboratory, where they were held in a cold room at 4 °C. Samples were shipped by courier under a USDA APHIS-PPQ permit (issued to J.K. Brown) to School of Plant Sciences, The University of Arizona, Tucson, AZ, USA.

### 2.2. Total DNA Isolation and PCR Amplification 

The glycerol was removed from cacao leaves by gently wiping each side with a kimwipe. The petiole and the leaf midvein and leaf lamina from the basal portion of each leaf were cut using a sterile razor blade and weighed. Samples (100 mg) were transferred to a 2 mL microfuge tube containing 2–3 metallic beads and stored at −80 °C. The total nucleic acids were isolated using a previously published cetyltrimethylammonium bromide (CTAB) method [23] and held at −20 °C. An aliquot of total DNA isolated was analyzed on an agarose (1%) gel by electrophoresis in Tris-Acetate buffer, pH 8.0 (100 V, 60 min). The nucleic acid bands were visualized by staining with GelRed (Gold Biotechnology^®^, Saint Louis, MO, USA) under ultraviolet light. The cloned insert was subjected to PCR amplification using primers pairs, CSSD 1–4 (Table 1), that amplified a fragment of the *mp* gene sequence and range in length from 400 to 1000 bp, respectively [8,22].

The PCR amplification reactions were carried out in a 25 μL volume containing 12.5 μL 2× Jumpstart RedTaq ReadyMix (Sigma-Aldrich, Saint Louis, MO, USA), 0.5 μL of 1.0 μM forward and reverse primer each, 5 μL of total DNA template (at variable initial concentrations of 20–30 ng/μL), and 6.5 μL of nuclease-free water. Cycling conditions were according to the previously published protocol [22]. Briefly, an initial denaturation step was carried out for 2 min at 94 °C, followed by 35 cycles of denaturation at 94 °C for 20 s, annealing at 50 °C (CSSD-1) or 55 °C (CSSD-2 through CSSD-4) for 20 s. The extension cycle was carried out at 72 °C for 30 s for CSSD-1 and 1 min for the CSSD 2-CSSD 4 primers. The final extension step was carried out at 72 °C for 10 min and 4 °C for 10 min, with a final hold at 12 °C.

### 2.3. Cloning and DNA Sequencing 

The PCR amplicons were ligated into the pGEM-T Easy plasmid vector (Promega, Madison, WI, USA) and transformed into *Escherichia coli* DH5α competent cells, using standard molecular biology protocols [24]. The presence of a cloned insert was determined by colony PCR amplification [25] using M13 universal primers (Promega Corporation, Madison, WI, USA). Two clones per amplicon were subjected to bidirectional capillary DNA sequencing (Sanger) carried out at Eton Biosciences (San Diego, CA, USA). The electropherograms were inspected and sequences of suitable quality were trimmed and assembled using Geneious Prime v2021.2 software (https://www.geneious.com low-quality sequences were removed. The *mp* sequences included in the final analyses (Table 2) were verified as badnavirus-like in an initial BLASTn search [26] of the nonredundant (nr) database available in GenBank (https://blast.ncbi.nlm.nih.gov/Blast.cgi?PAGE_TYPE=BlastSearch (accessed on 12 April 2021)). 

### 2.4. Pairwise Nucleotide Identity and Phylogenetic Analysis

Representative CSSD badnavirus reference sequences were downloaded from GenBank and trimmed to the length of the respective group-specific *mp* fragment. To calculate the percent shared nucleotide identity and establish provisional species identification, the partial *mp* sequences determined from field samples and reference sequences were subjected to pairwise distance analysis using the Sequence Demarcation Tool (SDT) v. 1.2 [27]. 

For phylogenetic analysis, sequences were aligned with representative reference sequences available in the GenBank using the MUSCLE algorithm [28]. The phylogeny was reconstructed using the maximum likelihood (ML) method (1000 bootstrap iterations) implemented in RAxML [29] using the best-fitting evolutionary model, which was identified as GTR gamma [30]. The ML analysis was carried out at the CIPRES web portal [31]. The phylogenetic tree was drawn and edited in FigTree software v. 1.4 (https://github.com/rambaut/figtree/releases (accessed on 1 December 2021)) and Inkscape (https://inkscape.org/pt/ (accessed on 1 December 2021)). 

### 2.5. Distribution Map of Badnavirus Species and Diversity Index

The spatial distribution map for the sampling sites was created with QGIS software version 3.16.8-Hannover (https://www.gnu.org/licenses/ (accessed on 15 April 2021)). Heat maps were produced with the QGIS heat map algorithm based on the location of the sampling site and the CSSD badnaviruses species identified, respectively. The radius and maximum values were calibrated to match the observed data. The algorithm predicted by the heat map outputs was based on the number of trees (points) sampled per location, such that the greater the number of points per defined region, the higher the map density. The diversity distribution was determined using DIVA-GIS 7.50 software by calculating the Brillouin index, based on the equation $=\frac{\ln N!-\sum \ln n_{i}!}{N}$, where H = diversity of species in a sample, *N* = total number of observations, and *n_i_* = number of individuals in the *i*-th class [32].

## 3. Results

### 3.1. Frequency of CSSD Badnavirus Detection

Cumulatively, the detection efficiency of the CSSD-1 primer pair, designed to amplify the known CSSD virus species, had the highest efficiency in terms of detection of the predominating cacao swollen shoot Togo B virus (CSSTBV), cacao swollen shoot Côte d’Ivoire E virus (CSSCEV), cacao swollen shoot Côte d’Ivoire D virus (CSSCDV), and cacao swollen shoot Togo A virus (CSSTAV) at 66.91% of the total symptomatic infected cacao samples collectively assessed in both countries (Table 2). In contrast, detection of CSSD viruses with the other three primer pairs, respectively, was 8.01% for CSSD-4, 5.19% for CSSD-2, and 0.15%) for CSSD-3 (Table 2). The primer pair had the capacity of amplifying mixed infection, which was revealed in amplicon sequencing and phylogenetic analyses (Table 2).

### 3.2. Prevalence and Diversity of CSSD Badnavirus Species

**Figure 2:**
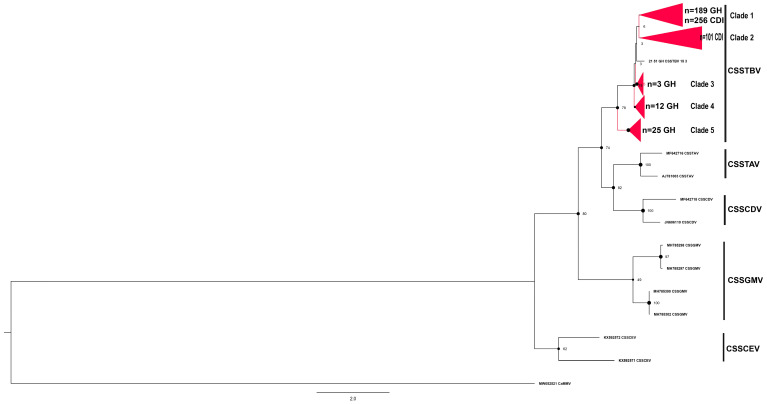
Maximum likelihood phylogenetic tree (1000 bootstrap iterations; ≥70% bootstrap support) reconstructed for the partial movement protein gene (*mp*) sequences of cacao swollen shoot Togo-B virus (CSSTBV) determined from cacao leaf samples collected in Côte d’Ivoire (CDI) and Ghana (GH) in this study (*n* = 588, red color) and representative CSSD badnavirus sequences available in the NCBI GenBank database. The CSSTBV partial *mp* region is located between nucleotide coordinates ~1780 and 2865 on the full-length genome. The tree was rooted with the New World cacao mild mosaic virus (CaMMV), GenBank Accession no. MW052521.

**Figure 3:**
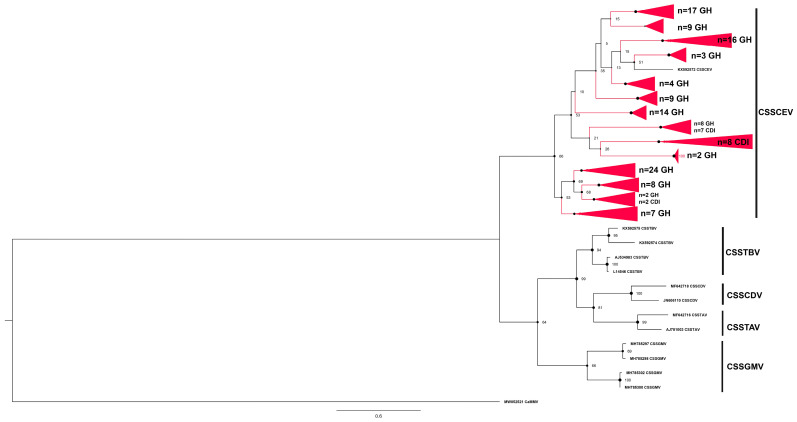
Maximum likelihood phylogenetic tree (1000 bootstrap iterations; ≥70% bootstrap support) reconstructed for the partial movement protein gene (*mp*) sequences of cacao swollen shoot CE virus (CSSCEV) determined from cacao leaf samples collected in Côte d’Ivoire (CDI) and Ghana (GH) in this study (*n* = 124, red color) and representative CSSD badnavirus sequences available in the NCBI GenBank database. For CSSCEV, the partial *mp* region is located between nucleotide coordinates ~1785 and 2900 on the full-length genome. The tree was rooted with the New World cacao mild mosaic virus (CaMMV), GenBank Accession no. MW052521.

**Figure 4:**
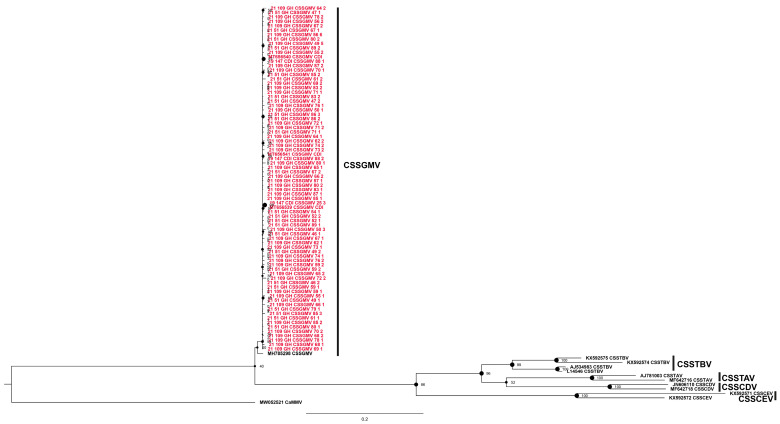
Maximum likelihood phylogenetic tree (1000 bootstrap iterations; >70% bootstrap support) reconstructed for the partial movement protein gene (mp) sequences of cacao swollen shoot Ghana M virus (CSSGMV) determined from cacao leaf samples collected in Côte d’Ivoire (CDI) and Ghana (GH) in this study (*n* = 78, red color) and representative CSSD badnavirus sequences available in the NCBI GenBank database. The CSSGMV partial *mp* region is located between nucleotide coordinates ~1850 and 2920 on the full-length genome. The tree was rooted with the New World cacao mild mosaic virus (CaMMV), GenBank Accession no. MW052521.

**Figure 5:**
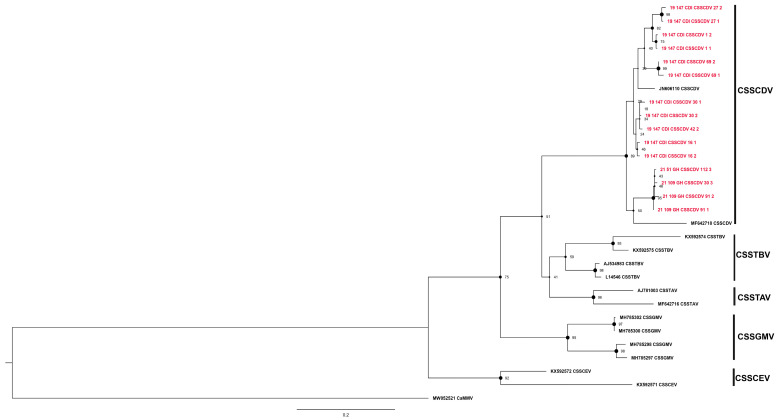
Maximum likelihood phylogenetic tree (1000 bootstrap iterations; >70% bootstrap support) reconstructed for the partial movement protein gene (mp) sequences of cacao swollen shoot CD virus (CSSCDV) determined from cacao leaf samples collected in Côte d’Ivoire (CDI) and Ghana (GH) in this study (*n* = 15, red color) and representative CSSD badnavirus sequences available in the NCBI GenBank database. For CSSCDV, the partial *mp* region is located between nucleotide coordinates ~1800 and 2200 on the full-length genome. The tree was rooted with the New World cacao mild mosaic virus (CaMMV), GenBank Accession no. MW052521.

**Figure 6:**
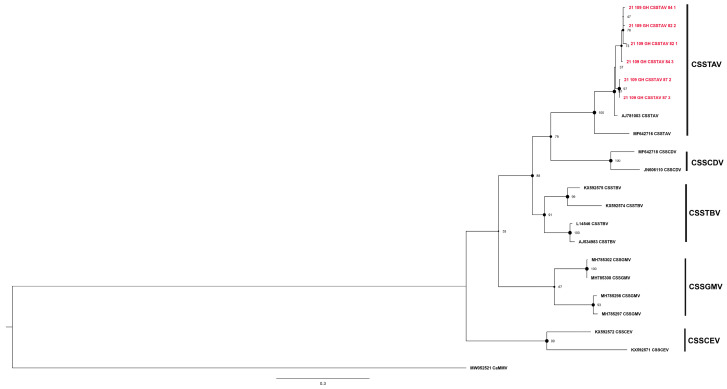
Maximum likelihood phylogenetic tree (1000 bootstrap iterations; >70% bootstrap support) reconstructed for the partial movement protein gene (mp) sequences of cacao swollen shoot Togo A virus (CSSGTAV) determined from cacao leaf samples collected in Ghana (GH) in this study (*n* = 6, red color) and representative CSSD badnavirus sequences available in the NCBI GenBank database. The CSSTAV partial *mp* region is located between nucleotide coordinates ~1925 and 3095 on the full-length genome. The tree was rooted with the New World cacao mild mosaic virus (CaMMV), GenBank Accession no. MW052521.

The partial *mp* sequences representative of each clade or sister clade (Figure 2, Figure 3, Figure 4, Figure 5 and Figure 6) generated from the study were submitted to the NCBI GenBank database and were assigned the accession numbers from OQ230632 to OQ230634 and from OQ305633 to OQ305812 (*n* = 183, Supplementary file S1). The cacao swollen shoot Togo-B virus (CSSTBV) species was the most prevalent CSSD badnavirus detected, comprising 588 sequences determined from 356 distinct CSSTBV-positive samples (Figure 2, Table 2). The CSSTBV species was equally represented among samples from Côte d’Ivoire and Ghana and was the predominant CSSD badnavirus species detected in cacao trees in both countries. The CSSTBV was evenly distributed in most sampling sites in Côte d’Ivoire and Ghana. Compared to the *mp* sequence of the isolates from Côte d’Ivoire that exhibited low sequence variability, several more highly divergent CSSTBV isolates were identified in Ghana. The highly divergent isolates were represented by 3 partial *mp* sequences from the Ashanti region (clade 3), 12 from the Eastern region (clade 4), and 25 from the Volta region (clade 5). Support for the latter subclades was moderate, with a bootstrap value of 78%. Based on the reference sequences with which they grouped, the latter variants were most closely related to CSSTBV isolates, previously reported to be predicted recombinants. These unique CSSTBV variants were all detected in one region, among the three adjacent cacao-growing regions of Ghana (Figure 2). Based on pairwise distance analysis, CSSTBV *mp* sequences exhibited moderate to very low variability at ~79–99% nucleotide identity (Supplementary file S2).

The cacao swollen shoot CE virus (CSSCEV) species represented the second most prevalent species, for which 124 partial *mp* sequences were determined from 71 positive samples (Figure 3, Table 2). The CSSCEV isolates harbored the most extensive *mp* sequence divergence among the five CSSD virus species identified in this study. Several highly divergent CSSCEV isolates from Côte d’Ivoire and Ghana grouped uniquely, albeit with low bootstrap support, from the other CSSCEV isolates clustering together as sister clades, respectively (Figure 3). The *mp* sequence divergence among CSSCEV isolates was consistent with the shared pairwise nucleotide identities ranging from 70.8% to 99.6% pairwise distances (Supplementary file S3).

The cacao swollen shoot Ghana M virus (CSSGMV) species was identified from 50 positive samples that yielded 78 sequences (Figure 4, Table 2), with isolates distributed relatively evenly among the collective study sites in Côte d’Ivoire and Ghana. Based on phylogenetic analysis, all the CSSGMV isolates were grouped into one clade, with robust bootstrap support. Based on pairwise distance (SDT) analysis, the 78 CSSGMV sequences shared 99–100% nucleotide identity, a result that was consistent with the relationships reconstructed in the phylogenetic tree. The CSSGMV isolates from Côte d’Ivoire and Ghana shared ~83–97% nucleotide identity with two previously reported sister clades comprising isolates from Nigeria, which appear to be endemic to southern Nigeria [7] (Figure 4; Supplementary file S4) for the disease was first reported there as well as the distinctive red vein-banding symptoms described (see references in [7]). Cloning and sequencing of the genome for nine Nigerian isolates led to the recognition of a new species, cacao red vein-banding virus [7]. The name was revised by the ICTV Badnavirus working group that assigned CSSGMV as the species name, which obliterated reference to the distinctive symptom phenotype that best sets this species apart from other CSSD virus species. 

The cacao swollen shoot CD virus (CSSCDV) species was detected in nine cacao samples from Côte d’Ivoire and Ghana that yielded 15 *mp* sequences, and CSSCDV was less prevalent compared to the three species reported above (Table 2). Phylogenetic analysis indicated that the CSSCDV isolates from Ghana clustered in a clade basal to the sister clade containing isolates from Côte d’Ivoire, with reasonably robust bootstrap support, at 89%. Overall, divergence of the CSSCDV *mp* sequences from Côte d’Ivoire was greater than for CSSCDV isolates from Ghana (Figure 5; Supplementary file S5). Based on pairwise distance analysis CSSCDV Ghana isolates shared ~98–99% nucleotide identity with each other, while CSSDV Côte d’Ivoire were ~91–99% identical. Finally, the sister clade from Ghana shared only ~91–93% nucleotide identity with isolates from Côte d’Ivoire (Supplementary file S5), a pattern that is indicative of that geographic isolation.

In Ghana, cacao swollen shoot Togo-A virus (CSSTAV) was detected in only three samples, yielding six sequences (Table 2) collected in Ghana. These isolates were detected in trees on a farm in Ghana located in Oti, near the Ghana-Togo border (Figure 6, Table 2). They formed a robustly supported sister clade, with a 97% bootstrap value, with a previously reported CSSTAV isolate from Togo (AJ781003) (Figure 6). Collectively, the CSSTAV sequences shared ~96–99% nucleotide identity (Supplementary file S6).

### 3.3. Geographic Distribution of CSSD Badnavirus Species

**Figure 7:**
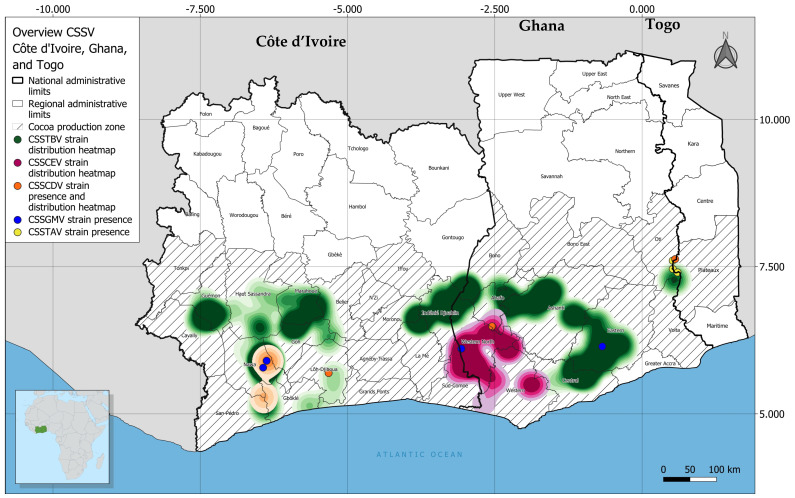
Heatmap of geographic distribution of different badnavirus species in Côte d’Ivoire and Ghana. The provisional species detected in Côte d’Ivoire were cacao swollen shoot CD virus (CSSCDV), cacao swollen shoot CE virus (CSSCEV), cacao swollen shoot Ghana M virus (CSSGMV), and cacao swollen shoot Togo B virus (CSSTBV). In Ghana, the provisional species detected were cacao swollen shoot CD virus (CSSCDV), cacao swollen shoot CE virus (CSSCEV), cacao swollen shoot Ghana M virus (CSSGMV), cacao swollen shoot Togo A virus (CSSTAV), and cacao swollen shoot Togo B virus (CSSTBV).

**Figure 8:**
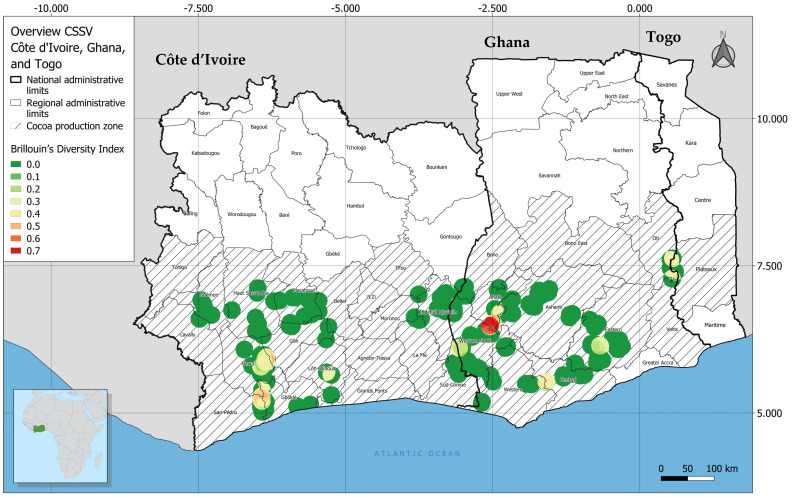
Map showing the diversity of identified CSSD badnavirus species based on the Brillouin diversity index [33]. In Côte d’Ivoire, four species were identified, cacao swollen shoot CD virus (CSSCDV), cacao swollen shoot CE virus (CSSCEV), cacao swollen shoot Ghana M virus (CSSGMV), and cacao swollen shoot Togo B virus (CSSTBV). In Ghana, the five species identified were cacao swollen shoot CD virus (CSSCDV), cacao swollen shoot CE virus (CSSCEV), cacao swollen shoot Ghana M virus (CSSGMV), cacao swollen shoot Togo A virus (CSSTAV), and cacao swollen shoot Togo B virus (CSSTBV).

Among the five CSSD badnavirus species identified in Cote d’Ivoire and Ghana, CSSTBV was the most prevalent and widely distributed species (Figure 7). In Côte d’Ivoire, CSSTBV was highly prevalent in Cavally, Guémon, Haut Sassandra, Marahoué, Gôh, Nawa, and San-Pédro, which are located in the southwestern and central regions of the country. Also, CSSTBV was detected in Indénié-Djuablin, Moronou, and La Mé cacao-growing regions in eastern Côte d’Ivoire. In Ghana, CSSTBV was prevalent and widely distributed on farms in the Bono, Central, Ashanti, Ahafo, Eastern, and Oti regions that collectively share a border with Togo. The second most prevalent species was CSSCEV. This virus was detected in farms along the western border of Ghana that is adjacent to the Western North, Western South (Ghana), and Sud-Comoé (Côte d’Ivoire) regions of Côte d’Ivoire. In Côte d’Ivoire and Ghana, the prevalence of CSSCDV, CSSGMV, and CSSTAV was lower than CSSTBV or CSSEV prevalence. Only sporadic CSSDV infection of trees was detected in Nawa, San-Pédro, and Lôh-Djiboua, which are in the southwestern Côte d’Ivoire. In Ghana, detection of CSSDV was rare and infected trees were limited to the Oti Region along the eastern border, where it frequently occurred in mixed infections with other CSSD badnaviruses. Only three trees were found to be infected by CSSTAV and they were found in the Oti region of Ghana, near the Togo border (Figure 7). Finally, CSSCEV was detected on farms located primarily in the Western Ahafo region of Ghana (Figure 8). Among the CSSD virus species identified in Ghana, the Brillouin diversity index was the highest for CSSCEV at 0.7, the species that is thought to have only recently emerged as a member of the CSSD species complex in West Africa [8,22].

## 4. Discussion

In this study, the *mp* region of CSSD badnaviruses was used as an informative marker to determine the detection frequency and map the geographic distribution of the key CSSD badnavirus species circulating in the affected cacao plantation in Ghana and Côte d’Ivoire. This was achieved based on PCR amplification with previously reported primer pairs, i.e., CSSD 1–4 primer pairs, and the resultant amplicon sequences to establish provisional species identification and diversity [22,34]. This extensive surveillance program was undertaken to map the phylogeographical distribution and better understand the phylodynamics of the CSSD badnavirus complex associated with commercial trees in major cacao-producing regions of Côte d’Ivoire and Ghana. These results build on previous studies that have also used PCR amplification of a fragment of the *mp* for CSSD virus detection and provisional species identification; however, in those studies, fewer samples were collected and analyzed over a narrower geographical area, respectively [21,22,34]. Collectively, results presented in this study have corroborated the extensive variability inherent to the CSSD badnavirus *mp* coding region as was previously reported and known to be flanked by conserved sequences [22]. These features have facilitated the design of degenerate primers that reliably detect the five apparently most prevalent CSSD badnavirus species in West Africa [22]. 

The CSSD-1 primers yielded the highest frequency of virus detection at 66.91%, which detected CSSCDV, CSSEV, CSSTBV, and CSSTAV in symptomatic cacao trees. The CSSD-4 primers yielded the second highest detection frequency of 8.01% and detected only the CSSGMV species, which is consistent with primer design based on this single, divergent species [22]. The CSSD-2 primers detected CSSD viruses in 5.19% of cacao samples, identified as CSSTAV and CSSTBV, with several isolates having as their closest relatives those previously identified as predicted CSSTBV recombinants [8]. Unexpectedly, the CSSD-3 *mp* primers, which were specifically designed to detect CSSCEV, the most recently emergent species [6,22], detected CSSCEV in only one sample, whereas the CSSD-1 primers detected all of the other CSSCEV variants identified in this study.

Recent sequencing efforts in several laboratories have produced a substantial number of CSSD badnavirus genome sequences from symptomatic cacao trees in West Africa and, collectively, have shown that there are at least five apparently predominant badnavirus species associated with CSSD of cacao. The striking inter- and intra-specific genomic sequence divergence among these five species provide potential clues about the approximate time of their emergence in cacao and, possibly, this divergence might be due to the occurrence of different primary and other alternative host species [8,12]. In addition to these five badnaviruses, five far less predominant species have been identified in cacao germplasm collections and/or commercial cacao trees elsewhere [5,34]. However, the importance of the latter species to commercial cacao production has not been studied. 

In this study, among the five CSSD badnavirus species detected, CSSTBV was the most prevalent and widespread (*n* = 406; Table 2). It was among the first two CSSD badnaviruses discovered and for which the genome sequence was determined, leading to the expectation that CSSTBV might be the most highly prevalent and widely distributed cacao-infecting CSSD species in West Africa [8,19]. The surveillance results reported here are consistent with the latter hypotheses, in that CSSTBV was identified as the most widely distributed and highly prevalent CSSD badnavirus species in Côte d’Ivoire and Ghana. Also, the CSSTBV *mp* fragment sequences exhibited the lowest divergence among the five CSSD species, a genome-associated feature that further supports its early emergence in cacao after the introduction of cacao as a nonendemic crop in West Africa (Supplementary file S2). Strikingly, the symptomatic trees in which CSSTBV was detected exhibited severe foliar and swelling symptoms and suffered the greatest yield/pod loss in both Côte d’Ivoire and Ghana. These observations support the hypothesis that CSSTBV was either the first or one of the first species to emerge from endemic wild host plant(s) and establish in commercial cacao farms, first in Ghana and then Côte d’Ivoire [35,36,37,38]. The second most prevalent species, CSSCEV, was detected primarily in trees on farms located along the border shared by Côte d’Ivoire–Ghana (Figure 7, Table 2). The genome sequences of CSSCEV isolates have been previously reported to harbor extensive variability, which has been considered reminiscent of very recent emergence [8]. This hypothesis is consistent with the extensive variability observed among the CSSCEV *mp* sequences determined here, which exhibit extreme intraspecific divergence (Supplementary file S3). The results further underscore the contribution of the extensive genomic variability harbored by these geographically restricted CSSCEV isolates and pinpoints the origin of the species near the border between the two countries where forests have been recently partially cleared for cacao production (Figure 3; Supplementary file S3). 

Further, it was in western Ghana where the initial unusual foliar symptoms and unique swellings on branches were first observed in ~2000, followed by tree decline within one year after symptom development, and where outbreaks are now attributable to CSSCEV. Thus, CSSCEV appears to have emerged from several native plant/forest tree species there, where studies have previously identified endemic non-cacao plant species as CSSD virus hosts/reservoirs [39,40]. In this study, the prevalence and distribution of CSSGMV in Côte d’Ivoire (Figure 7, Table 2) was relatively limited compared to the other four CSSD badnavirus species detected. In contrast, in Ghana, CSSGMV prevalence was sporadic and, when it was detected, the virus occurred primarily in mixed infections with other CSSD species. Unexpectedly, the prevalence of CSSCDV in Côte d’Ivoire was high and the virus was broadly distributed, as has been reported in previous studies [5,14,19,34]. In contrast, in Ghana, the prevalence of CSSCDV was few and the distribution was limited, as was revealed in the present study. These observations suggest that CSSCDV may have originated in Côte d’Ivoire, from where it has been recently dispersed to Ghana either by the mealybug vector and/or budwood. Finally, the distribution of CSSTAV was limited to the Ghana–Togo boarder, suggesting that this species may be more prevalent in Togo, where the disease is known to occur but for which species prevalence and distribution information is mostly unavailable [41]. The minimal information about CSSD genomic variability/diversity and its occurrence in Togo indicates future surveillance is needed to map the countrywide distribution of CSSTAV there and to determine the likelihood of regional spread, especially into CSSTAV-free regions of Côte d’Ivoire and Ghana (this report) and likely Nigeria [7].

Improved understanding of CSSD badnavirus species and genomic diversity as revealed in this study indicates uneven prevalence of CSSD virus species in Côte d’Ivoire and Ghana and thus highlights the importance of regional surveillance extended beyond individual countries. Additional knowledge in this regard is required to guide broader disease management based on epidemiological data obtained using a standardized molecular diagnostic for the badnavirus species circulating in cacao plantations. In this regard, further expansion of the field surveillance and mapping of the predominant CSSD species associated with cacao plantations as well as the key alternate/wild CSSD virus hosts in the other West African countries such as Togo and Nigeria are, thus, highly recommended. This could help identify important reservoirs of the different CSSD species and illuminate the potential for spread/dispersal of CSSD species into uninfected and/or newly planted cacao plantations in West Africa. The information would also be valuable for pinpointing the center(s) of origin and diversification of CSSD badnavirus species throughout West Africa and primary reservoirs of extant and currently unknown CSSD virus species hosted in cacao plants and alternative wild trees and weed species. Also, improved molecular surveillance methodologies to map mealybug vector–virus species co-distributions would fill present gaps in knowledge about badnavirus-vector dynamics in the West African cacao producing region [36,38,40].

In conclusion, the updated knowledge of CSSD badnavirus species diversification, prevalence, and distribution as presented in this paper was facilitated by the joint molecular surveillance in Côte d’Ivoire and Ghana, which are known to be the two largest cacao-producing countries in West Africa. The results showed that primer CSSD-1 detected four of the five predominant CSSD-associated badnaviruses in symptomatic cacao trees. Using CSSD-1, 66.9% samples tested positive, while CSSD-2, 3, and 4 primers revealed CSSD badnaviruses in 8%, 5%, and 0.15% of samples (Section 3.1 of Results, Table 2). Further, the results showed that the CSSD1–4 diagnostic primer pairs were capable of detecting five apparently predominant CSSD virus species actively circulating in symptomatic cacao trees, making them highly amenable for routine badnavirus surveillance in commercial cacao production throughout the region. These results provide further evidence that the CSSD1–4 diagnostic primer pairs could detect the main predominant CSSD virus species circulating in symptomatic cacao trees and, thus, could be adopted for routine field surveillance activities. These observations are consistent with previous reports that have demonstrated the robustness of the *mp* coding region as an informative marker for detection and differentiation of multiple CSSD virus species prevailing in field infections [18,19,22]. Finally, validation of the CSSD 1–4 primers for early detection of the five most predominant badnaviruses in asymptomatic cacao trees and/or in their wild host plant species, could lead to the expanded use of these primers for the detection of latent infections in cacao, as well as the discovery of asymptomatic/symptomatic wild forest trees and/or of alternative hosts. Collectively, this new knowledge together with a robust molecular detection tool would provide the necessary support to cacao breeding programs in identifying species-specific and/or broad-species CSSD resistance to aid in long-term management of the disease through screening of virus-free planting materials (cacao clones) prior to distribution and planting in farmers’ fields. 

## Figures and Tables

**Table 1:** Primers used to amplify the partial movement protein sequence of cacao swollen-shoot-disease-associated badnaviruses from West Africa [22].

Badnavirus Species Acronym *	Primer Designation	Forward (F) and Reverse (R) Primer Sequence Based on a 5′ to 3′ Orientation	Tm°C	Amplicon Size in Base Pairs (bp)
CSSD-1	CSSD1_F	AAYTGGCARAAYGGAGARGC	50	~400 bp
CSSTBV CSSCDV CSSCEV	CSSD1_R	CTTCYTCYCCAATTATCCAGACTGC		
CSSD-2	CSSD2_F	ATGCAACCHARRTCGGTWGAAAC	55	~850 bp
CSSTBV CSSCDV	CSSD2_F	TCYATYTTYTCTGTTGGGTCCG		
CSSD-3	CSSD3_F	AGTCAAAGGGGAAGRSAACC	55	~700 bp
CSSCEV	CSSD3_R	CCRTTYTGCCARTTNTCRTAYCC		
CSSD-4	CSSD4_F	AATCACAAGAAGTATGACAGGGAG	55	~1000 bp
CSSGMV	CSSD4_R	TTCATTCGCCATTGTATCCAC		

* Legend: cacao swollen shoot CD virus (CSSCDV) [5], cacao swollen shoot CE virus (CSSCEV), previously, cacao red vein virus (CRVV) [6], cacao swollen shoot Ghana M virus (CSSGMV), previously cacao red vein banding virus (CRVBV) [7], and cacao swollen shoot Togo B virus (CSSTBV), previously cacao swollen shoot virus (CSSV) [13,14].

**Table 2:** Frequency of PCR detection of cacao swollen shoot (CSSD) badnaviruses identified based on sequence analysis of a fragment of the movement protein (mp) gene from symptomatic cacao leaf/petiole samples collected in Côte d’Ivoire and Ghana.

Collection No.	Sample Location, by Country	Total No. Samples Analyzed by ^1^ PCR Amplification	Primers Used for Amplification of Badnavirus Cacao Swollen Shoot Disease (CSSD) and Detection Frequency			
			CSSD-1 Primers Positive	CSSD-2 Primers Positive	CSSD-3 Primers Positive	CSSD-4 Primers Positive
1	Côte d’Ivoire I	100	38 CSSTBV = 34 CSSCDV = 3 ^2^ Mixture * = 1	1 ^3^ NC = 1	0	2 CSSGMV = 2
2	Côte d’Ivoire II	185	149 CSSTBV = 143 CSSCEV= 6	8 CSSTBV = 8	0	2 CSSGMV = 2
3	Ghana I ^4^ WR	120	84 CSSTBV = 6 CSSCEV = 63 cacao host = 2 NC = 13	0	0	2 CSSGMV = 1 Mixture ** = 1
4	Ghana II ASH, BR, ER, CR	178	146 CSSTBV = 125 CSSCDV = 4 Mixture *** = 10 host amplicon = 5 NC = 2	14 CSSTBV = 10 Mixture *** = 3 NC = 1	0	24 CSSGMV = 1 Mixture *** = 12 NC = 11
5	Ghana III ER, VR	91	34 CSSTBV = 24 CSSTAV = 2 Mixture * = 1 Mixture *** = 5 host amplicon = 3	12 CSSTBV = 6 CSSTAV = 1 Mixture *** = 1 NC = 4	1 CSSCEV = 1	24 CSSGMV = 6 Mixture *** = 17 Mixture **** = 1
Total		674	451	35	1	54
**Percent badnavirus-positive** **cacao leaf samples amplified**			**66.91**	**5.19**	**0.15**	**8.01**

Legend: ^1^ PCR = polymerase chain reaction. ^2^ Mixed infections denoted as: * mixed infection of CSSTBV and CSSCDV (amplified by CSSD1); ** mixed infection of CSSCEV and CSSGMV (amplified by CSSD1 and CSSD-4, respectively); *** mixed infection by CSSTBV and CSSGMV (amplified by CSSD1 and CSSD-4, respectively); **** mixed infection by CSSTAV and CSSGMV (amplified by CSSD2 and CSSD-4, respectively). **^3^** NC = not cloned; unable to clone the amplicon. ^4^ Location abbreviations: ASH = Ashanti Region, BR = Bono Region, CR = Central Region, ER = Eastern Region, VR = Volta Region, and WR = Western Region.

## Data Availability

The sequence data or data used to construct the maps are available from the corresponding authors upon reasonable request. Representative sequences have been submitted to the NCBI GenBank database.

## Supplementary Materials

The following supporting information can be downloaded at: https://www.mdpi.com/article/10.3390/v16050735/s1, file S1: GenBank_accessions, file S2: CSSTBV_SDT_data, file S3: CSSCEV_SDT_data, file S4: CSSGMV_SDT_data, file S5: CSSCDV_SDT_data, file S6: CSSTAV_SDT_data.
